# The triple helix in developed countries: when knowledge meets innovation?^☆^

**DOI:** 10.1016/j.heliyon.2022.e10168

**Published:** 2022-08-12

**Authors:** Filip Fidanoski, Kiril Simeonovski, Tamara Kaftandzieva, Marina Ranga, Leo-Paul Dana, Milivoje Davidovic, Magdalena Ziolo, Bruno S. Sergi

**Affiliations:** a UNSW Business School, University of New South Wales, Australia; b Independent Scholar, Macedonia; c Ss. Cyril and Methodius University in Skopje, Macedonia; d Joint Research Centre, European Commission, Spain; e Rowe School of Business, Dalhousie University, Canada; f Finance Academic Group, Northeastern University, USA; g University of Szczecin, Poland; h Harvard University, USA & University of Messina, Italy

**Keywords:** Data envelopment analysis, Efficiency, Triple helix model of innovations

## Abstract

This paper deals with innovation viewed through the triple helix model as a milestone in the contemporary society of knowledge-based economies. Our goal is to empirically investigate the (in)efficient utilisation of academia, industry and government as three helices in order to boost innovations. Therefore, we construct a sample of 30 developed OECD countries with data covering the period from 2006 to 2018 and set up an input-oriented BCC data envelopment analysis that employs variables with non-negative average values over the entire period to calculate their efficiency scores. Our estimates from the radial models show that countries could reduce their inputs by a mean value of 11.9 per cent and keep their level of innovations in the triple helix model and by a mean of 5.8 per cent on average in the extended quintuple helix model. We find higher total inefficiencies in the non-radial models, which amount to 25.3 per cent on average in the triple helix model and 21.8 per cent on average in the quintuple helix model. The breakdown of the inefficiency score for different inputs reveals that countries have the largest potential for reducing CO_2_ emissions and the least room to reduce the Education Index and Civil Society Participation.

## 1 Introduction

Society has been experiencing various changes and facing multiple challenges in recent decades. Knowledge becomes the most challenging imperative for any policymaker. Correspondingly, a growing body of literature addresses the interaction between universities, industries, and governments. The concept of networking is not new in practice. Modern academic language acknowledges this concept as a triple helix (TH). While Schumpeter's theory of creative destruction shows how outmoded economic regimes disappeared, the TH systems delineate how new regimes appear through inventive construction. By revealing the engine's workings, they provide new insights into the process of knowledge-based development that is often considered opaque and hidden, such insights encouraging initiatives and practices that carry the seeds of innovative developments (Ranga and Etzkowitz, 2013).

The triple helix theory provides a general paradigm, analytical framework, and method for studying the relationship between innovation actors at the system level (Strand et al., 2017). Furthermore, it is not pretentious or wrong to state that these actors play a crucial role in creating an entrepreneurial society. The underlying model differs from the other approaches and concepts, such as national innovation systems or the triangle model. From the human capital approach, we understand that education policies are essential in creating sustainable growth and development conditions; we witness very bright and capable people who change our mindsets and live through innovation. As for the increased salience of innovations, it is helpful to prepare a deeper and broader inquiry into the role of government and universities in entrepreneurship development. For that purpose, we have used the TH model in the case of the member countries of the Organisation for Economic Cooperation and Development (OECD). Specifically, TH is an analytical model that explains their dynamics in describing the variety of institutional arrangements and policy models (Etzkowitz and Leydesdorff, 2000).

This paper deals with the interaction between university, industry, and government. That is the key milestone and pillar for economic growth based on innovation and knowledge. In ancient Mesopotamia, a triple helix water screw, invented to raise water from one level to another, was the basis of a hydraulic system of agricultural innovation that irrigated ordinary farms as well as the Hanging Gardens of Babylon, one of the seven wonders of the ancient world. In contemporary times, the university is the generative principle of knowledge-based societies, just as government and industry were the primary institutions in industrial society. The transformation of a university from teaching to research and then to an entrepreneurial institution is a vital and emerging paradigm (Etzkowitz, 2008).

Nowadays, the university is receiving intensified attention for fostering entrepreneurship and filling the role of a mediator to enhance high-tech developments (Brem and Radziwon, 2017; Cai and Etzkowitz, 2020). The triple helix plays a crucial role in integrating the universities, firms, and governments whose close interaction and optimal collaboration enable nations to anticipate how they could create wealth and build a knowledge-based society (Sarpong et al., 2017). Having in mind that sustainable development and technological advancement can hardly be isolated one from another; humanity is facing a significant challenge in attaining a harmonious and mutually reinforcing dynamic between them both while minimising the adverse social and economic effects on a global scale: resource depletion, environmental degradation, escalating inequality and population explosion (Zhou and Etzkowitz, 2021). Therefore, it is important to assess the functioning of the TH system in the world. Theoretically, this paper presents a creative synthesis of the previous theoretical and empirical debates in this field. Various scholars have published studies about the interaction between different actors in innovation. The paper also stresses the importance of this system's earlier roots, origins, and precedents. We focus on the contemporary evolution of the TH concept and consider successful stories from the world.

A case in point, new knowledge increasingly appears in polyvalent forms and modes, with theoretical, practical, and interdisciplinary implications forming a common centre of gravity – the DNA of the TH (Viale and Etzkowitz, 2005). From an empirical point of view, our original intention is to define the university's characteristics, to understand the complex nature of the interaction between various actors in a knowledge society, their spillover effects, and the benefits from their activities. Importantly, we attempt to discover the triggers and obstacles to fostering innovation. We run the advanced multi-criteria decision-making methods by searching these linkages and modes. The results should be beneficial for the decision-makers to design and implement better policies to enhance the functioning of the economy and ensure the future of their citizens. The study will undoubtedly enrich the scope of academic knowledge about TH at a global level.

The paper is structured as follows. Section 2 is devoted to the TH model's previous theoretical and empirical findings. Section 3 explains the sample and defines the variables used in the model, while in Section 4 we report the sample's descriptive statistics. In Section 5 we describe the methodology for the analysis, and the results are presented and discussed in Section 6. The paper concludes with the final remarks made in Section 7.

## 2 Theoretical background and related literature

Studying the factors driving economic growth has received considerable attention, especially now when sustainability is the main focus of the scientific discourse, and many questions remain unanswered. Since technology transfer has not lived up to expectations and failed to lead to sustainable economic growth (Saad et al., 2008), it is evident that alternative sources of economic growth are required (Etzkowitz et al., 2007). Researchers have discovered that entrepreneurial activity and innovation are key drivers of economic growth. Consequently, industrial societies have gradually transitioned to knowledge-based, thus creating a new social order (Etzkowitz, 2002; Cáceres-Carrasco and Guzmán-Cuevas 2010; Galindo-Martin et al., 2010; Cai and Etzkowitz, 2020).

The core premise of fostering an innovation system lies in the holistic framework of reciprocal links between government, academia, and industry. The interconnection and interaction between the three pillars form the well-established Triple Helix Innovation Model (Etzkowitz and Leydesdorff, 2000). This model posits that industry, academia, and governments are the three helices of economic development. The knowledge spillover effect is created, transferred, and internalised through the interdependence and cooperation among those heterogeneous organisations (Zhuang et al., 2021). Each actor interacts and cooperates with other actors to enhance all involved sectors. The role of academia is to generate new knowledge and technology; the industry's role is to commercialise this knowledge and engage in production activities, while the government oversees and enforces it (Etzkowitz, 2003a). At this stage, most cooperative initiatives occur at the regional level (Smith and Bagchi-Sen, 2010). Such initiatives are usually geared toward filling gaps in economic development, addressing existing problems in industrial clusters, and solving the problem of inadequate governmental oversight.

Theories following the TH approach prove to some extent the idea that universities play a vital role in the innovation process (Cai and Etzkowitz, 2020) as far as knowledge-based and sustainability-oriented societies are concerned (Etzkowitz, 2003a, 2003b; Leydesdorff and Meyer, 2003; Etzkowitz and Dzisah, 2008; Cai and Etzkowitz, 2020). The TH model has a dual nature: it is an innovative system concept and a non-linear model of innovation. Non-linear models of innovation develop a linear approach by taking institutional inter-relations into account, thus changing the relationships between input and output (Etzkowitz and Leydesdorff, 2000; Balzat and Hanusch, 2004; Etzkowitz, 2008).

The university is less potent than the other two strands out of the three helices. However, the traditional boundaries of each of the three helices have overlapped, since each of the spheres has assumed the role of the others (Etzkowitz, 2008). Nowadays, triple helix interactions are increasingly taking place in transnational contexts. Social sciences and computer sciences are integrated using the Living Lab, machine learning techniques, and ongoing activities to predict future tendencies (Cai et al., 2019). Nevertheless, the Triple Helix model highlights the importance of the universities and provides them with a more prominent role in the innovation process. The university nowadays initiates diverse changes in the institutional environment and actively participates in implementing such changes (Cai and Liu, 2020). Thus, apart from its traditional role in providing a trained workforce and publications to society (Gibbons and Johnston 1974; Onida and Malerba 1989; Martinelli et al., 2008), the role of academia is broadened to encompass a greater focus on the practical aspects of research and engage in entrepreneurship, hence operating as a catalyst for the technological development from a prototype to the final concept (Etzkowitz and Leydesdorff, 2000; Etzkowitz and Klofsten, 2005; Etzkowitz et al., 2007; Audretsch et al., 2011; Cai and Etzkowitz, 2020). In the knowledge-based economy, the university is arriving at a standard entrepreneurial format that incorporates and transcends its traditional educational and research missions, thus fostering social development (Etzkowitz and Zhou, 2017). University's engagement in entrepreneurship or commercial activities usually refers to the generation, application, use, and exploitation of university knowledge and capabilities outside the university context and the marketplace (Molas-Gallart and Castro-Martínez, 2007). Scholars highlight universities' unique historical and social factors contributing to a sustained competitive advantage (O'Shea et al., 2007). Geoghegan et al. (2015) point out that each country's university commercialisation orientation exhibits further evolutionary development. Universities with more experience in technology transfer will generate more licenses and licence-generated income.

Every economic theory has its criticisms. The theory of the TH model is not different from the norm. One of the main criticisms of the model is that it is very abstract (Cooke, 2005; Martynovich, 2011). The Triple Helix model proposes maximum engagement and collaborative relationships between academia, industry, and government, but provides no practical directions on bridging differences and nurturing cooperation between the three spheres (Tuunainen, 2005; Lundberg, 2013). A closely related criticism is that the TH model does not consider the national setting, which influences the three institutional actors.

Additionally, the model does not consider the differences in different countries' innovation systems (Balzat and Hanusch, 2004). The effectiveness of this model has been questioned, as regions have failed to meet expected levels of innovation, GDP development, and employment (Asheim and Coenen, 2005; McAdam et al., 2012). The TH model has been criticised for lacking a solid micro-foundation and ignoring people who are, in the initial case, the main drivers of innovation (Brännback et al., 2008).

We agree that there have been a lot of studies on university-industry collaboration (Dosi et al., 2006; Lam, 2008). There has also been a lot of research into the relationship between industry and government (Feldman and Kelley, 2006). However, the exact nature and development of the relationship between university and government, and the trilateral relationship between university, industry, and government are still mostly unclear. One of the biggest challenges of the TH model is choosing a suitable measurable indicator that can serve as a proxy for the current innovation system (Martynovich, 2011).

Nevertheless, despite the current weaknesses, the significance of TH systems for advancing innovation and fostering sustainability is increasing, due to the global economic restructuring that requires a novel type of industry based on R&D-driven innovation and advanced technologies (Švarc, 2014). The triple helix perspective has enriched the conceptual and empirical dimensions of innovation as a systemic phenomenon, thus potentially improving the effectiveness of innovation policies at regional and national levels (Leydesdorff and Zawdie, 2010). Scholars adopt the TH model to develop sustainable solutions and niche innovation projects to solve global challenges (Audretsch and Link, 2012). The robust collaboration between the three spheres is becoming a key success factor for the growth of regional entrepreneurial ecosystems since it fosters innovation as an answer to key ecological problems (Brem and Radziwon, 2017).

Furthermore, innovation is a multilevel concept since national and regional innovation systems coexist, coevolve, and jointly form the framework for producing a country's innovation. The evaluation of the efficiency and performance of the innovation system remains a high priority in order to improve innovation policies and practices. A data envelopment analysis (DEA) methodology has been successfully applied to measure the efficiency of innovation systems on both national (Pan et al., 2010; Cullmann et al., 2011; Afzal, 2014; Tarnawska and Mavroeidis, 2015; Prokop and Stejskal, 2017; Anderson and Stejskal, 2019; Aguilar-Barceló and Higuera-Cota, 2019; De la Vega et al., 2019) and regional level (Zabala-Iturriagagoitia et al., 2007; Chen and Guan, 2012; Broekel et al., 2018; Fadeyi et al., 2019; Zemtsov and Kotsemir, 2019; Zhuang et al., 2021). When analysing the efficiency of innovation systems, the DEA methodology is usually used by examining the input-output components in order to model a robust efficiency measurement. The efficiency of innovation systems is defined as the maximisation of innovation outputs through effective internal resource allocation and system operation under the given factor inputs (Carayannis et al., 2016). Thus, DEA models focus precisely on the input-output efficiency of innovation systems, with each country or region considered an independent decision-making unit (DMU) (Cai, 2011).

Concretely, Carayannis et al. (2016) estimate the national and regional innovation systems, considering the multiple stages of the innovation process (knowledge creation and commercialisation) using the multi-objective linear program DEA. The proposed approach applies to 23 European countries and their 185 corresponding regions. The results indicate significant differences in the efficiency scores of the distinct stages and levels. Germany and Switzerland have a relatively high overall efficiency, whereas Hungary, Denmark, and the UK have the lowest overall innovation efficiency scores. However, the more a country pursues innovation generation, the higher the tendency to innovate, but the efficiency of diffusing innovation can differ irrespective of the innovation ranking, as evidenced by Sweden, the most innovative EU member state, ranking lowest on the efficiency scale, even as Bulgaria and Romania, the two modest innovators, are relatively efficient (Anderson and Stejskal, 2019). According to Prokop and Stejskal (2017), most of the European countries (EU-28) fail in their attempt to become knowledge or knowledge-based economies; they are not effective during the processes of knowledge creation, use, dissemination, and commercialisation nor are they effective in the processes of using determinants of the knowledge economy (mainly financing of research and development). Pan et al. (2010) found that Asian countries perform better than European countries. Among the 33 analysed countries Japan, South Korea, Russia, Iceland, Romania, Slovakia, and Slovenia are the most efficient countries. Cullman et al. (2011) highlighted the R&D efficiency differences among OECD countries and their relationship to their regulatory environment. They found that Sweden, Germany, and the United States are the best-performing countries. Out of the analysis carried out on 20 emerging and developed countries using the DEA Bootstrap technique, Afzal (2014) concluded that it is essential for policymakers to evaluate how their countries position themselves in national innovation system (NIS) input-output combinations in terms of achieved efficiency to other countries.

On the other hand, the Latin American and Caribbean economies generally lag behind North America and Europe in creating innovation-friendly environments mainly due to problems related to human capital formation, the conduct and impact of research, and institutional aspects (Aguilar-Barceló and Higuera-Cota, 2019). Nevertheless, in line with the findings of Anderson and Stejskal (2019), the strong innovation results of several countries, such as Chile and Colombia are not matched by their efficiency management (Aguilar-Barceló and Higuera-Cota, 2019). The DEA methodology applied to five selected South American countries between 1990 and 2018 showed that the dynamism of the development initiatives passes through the generation of dynamic interactions of all the helices. However, the inadequate balance between the knowledge subsystems (helices) would dismantle the circulation of knowledge that should be oriented to sustainable development (De la Vega et al., 2019). A variety of input and output variables were selected in the respective DEA analyses as proxies for the three helices to measure the efficiency of innovation systems. Moreover, the examined studies cover different periods and sample countries; thus, their results are also different and contradictory.

## 3 Sample and variables

For our empirical analysis, we construct a sample of 30 OECD member states with annual data from 2006 to 2018. Countries were selected based on their OECD membership throughout the entire time horizon. The complete list of countries in the sample is presented in Appendix 1.

On the side of the inputs in our model, we use the Education Index from the Human Development Reports as a proxy for academia, the industrial value added of GDP as a measure for industry, and the R&D expenditure of GDP as a measure for the government. The choice of these measures as inputs was primarily influenced by the interactions between the helices. For instance, the Education Index shows the readiness of the country's educational sector to supply the industry and government with qualified labour; the industrial value added of GDP measures the contribution of the industry to the country's GDP; and R&D expenditures of GDP measures the level of support which the government extends to research facilities in order to produce innovations. Apart from these variables which we use as inputs in the baseline triple helix model, we add the Civil Society Participation indicator as a composite measure for the soundness of the civil society as the fourth helix and the CO_**2**_ emissions per capita as a proxy for the environment as the fifth helix in the extended quintuple helix (QNH) model. Our output variable, which measures the level of innovativeness due to the interactions between the helices, is the total number of patent applications per 100,000 persons.

The data for the selected variables were collected from multiple sources, including World Bank's World Development Indicators (WDI) and GovData360 (GOV) databases, UNDP's Human Development Reports (HDR) and US Energy Information Administration (EIA).

A summary of the variables used in the model is shown in Table 1.

**Table 1:** Definition of variables.

Variable	Abbreviation	Unit	Note	Source
**Input variables**				
Education Index	EDI	index value from 0 to 100	measure for academia in interaction with industry	HDR
Industrial value added	IVA	per cent of GDP	measure for the industry in interaction with the government	WDI
R&D expenditure	RDE	per cent of GDP	measure for government in interaction with academia	WDI
Civil Society Participation	CSP	index value from 0 to 100	measure for civil society	GOV
CO_2_ emissions per capita	CO2	metric tones per capita	measure for environment	EIA/WDI∗
**Output variables**				
Patent applications	PAT	number per 100,000 persons	measure for innovation	GOV/WDI∗

Notes: The original index values for the Education Index and Civil Society Participation ranges from 0 to 1 but, for convenience, they are multiplied by 100. The symbol ∗ denotes own calculation based on data from the given sources.

## 4 Descriptive statistics

In this section, we make a brief overview of the summary statistics for the selected variables, and we additionally study the differences between countries and the changes over time. Descriptive statistics are shown in Appendix 2.

The average value of the Education Index for the entire period is 84.1 with a tendency of a monotonic increase from 80.9 in 2006 to 86.3 in 2017 and 2018, yet the growth rate has slowed by the end of the period. Germany has the highest mean value of 93.1, followed by Australia, Denmark, and Norway with mean values above 90 index points. Most OECD member states range from 80 to 90 index points, with Turkey and Mexico being outliers with mean values of 64.8 and 64.5, respectively.

Industrial value added averages 24.5 per cent of GDP and has been relatively constant over time with a slight decrease from 26.2 per cent in 2006 to 24.3 per cent in 2018. A similar tendency can be observed for individual countries, with Norway recording the highest average of 34.7 per cent, ahead of the Czech Republic, Mexico, and Slovakia above 30.0 per cent. In comparison, Luxembourg has the lowest mean with 11.8 per cent of GDP.

Government support for research and development has also been relatively constant during the analysed period, averaging 1.9 per cent of GDP. After the initial mean values of 1.7 per cent in 2006 and 2007, the share of R&D expenditure reached the long-term average of 1.9 per cent in 2008 and never went above 2.0 per cent of GDP. South Korea records the highest mean R&D expenditure with a mean of 3.6 per cent of GDP, followed by Sweden, Japan, Finland, and Switzerland with averages above 3.0 per cent; on the other side, Mexico's government has spent the least amount on research and development with only 0.4 per cent of GDP, while governments of Slovakia, Greece and Turkey have also earmarked amounts by less than 1.0 per cent of GDP.

The Civil Society Participation index averaged 72.9 points, with a declining level of participation over time. Namely, the mean index values around 74 index points in 2006–2012 have exhibited a downward movement afterwards, reaching the lowest mean of 70.1 in 2018. Amongst individual OECD member states, the variability of the participation levels is relatively high with a standard deviation of 23.0 index points. While most countries have values ranging from 60 to 80 index points, others are also in the other deciles. For instance, Switzerland, Denmark, Norway, Germany, Finland, and the United States have means above 90 points. In contrast, Turkey, Hungary, and Mexico have recorded the lowest participation in civil society, with mean values below 60 index points.

Regarding environmental protection, the level of CO_2_ emissions per capita averages 10.2 metric tonnes per capita. There was a significant decrease in the level of emissions in the period 2006–2014, when the annual mean of 11.4 metric tonnes in 2006 fell to 9.6 metric tonnes per ton per capita in 2014, and this level was maintained until the end of the analysed period. The variability of the CO_2_ emissions between countries is relatively high with a standard deviation of 4.8. A country with the highest CO_2_ emissions per capita is Luxembourg, with 21.5 metric tonnes on average, whereas Mexico has the lowest mean emissions of only 4.2 metric tonnes per capita.

Finally, the average innovativeness of the OECD member states in the analysed period measured through the patent applications is 57.3 patents per 100,000 persons. In general, there is a decreasing tendency in the number of patent applications over time as the annual mean of 63.6 patents in 2006 reduced to 55.4 patents per 100,000 persons in 2018. However, after the initial drop to the dipping mean of 54.1 in 2010, there was a temporal surge until 2014, when the level of innovativeness somewhat increased. The most innovative countries in the analysed period are South Korea with a mean value of 377.5 patents and Japan with a mean of 271.3 patents. In comparison, the least innovative country is Slovakia with only 4.6 patents per 100,000 persons on average.

## 5 Methodology

Our analysis aims to obtain efficiency scores regarding the TH innovation model for each country in the analysed period. Considering this goal and that the TH model has three input components that are *married* to that innovation as output, we follow the efficiency literature and employ the data envelopment analysis (DEA) using DMUs (Cooper et al., 2011). DEA is a non-parametric technique that, through linear programming, approximates the true but unknown technology without imposing any restriction on the sample distribution. DEA is a complex benchmarking technique that yields production possibilities set where efficient multi-criteria DMUs on this surface shape the frontier (Lafuente et al., 2016).

Concretely, we use the most popular methodology, which compared to parametric approaches, has several important advantages (Deng et al., 2007): (i) it is not necessary to find out the concrete form of production function and is with fewer restrictions; (ii) it is easier to deal with the case with multiple inputs and multiple outputs; (iii) the technological efficiency analysis enables the enterprises to find out which input is not efficiently utilised and to look for the best way to improve efficiency in addition to knowing the input efficiency of the evaluated structure in question compared to the most outstanding enterprises; and (iv) the non-parameter approach allows not only to arrive at a conclusion about the technical efficiency but also to calculate the economic efficiency, allocation efficiency and pure technology efficiency, which makes it possible to conduct an inclusive evaluation and should be regarded as a comprehensive assessment index of achievements.

Before setting up the optimisation problem in our empirical analysis, we establish a set of assumptions about the modelling environment.

Assumption 1 (Linearity) *The objective function of optimising using DEA is linear*.

This assumption implies that the optimisation is done using a linear programming technique. However, this may be problematic in practice because the objective function and constraints are expressed as fractions and are thus non-linear, requiring the optimisation problem to be formulated in a linear form.

Assumption 2 (Non-negativity) *The values of the inputs*$x_{i,n}$*and outputs*$y_{i,m}$*as well as the weights*$\lambda_{i}$*are non-negative*, i.e., $x_{i,n},y_{i,m},\lambda_{i}\geq 0$.

Non-negativity means that the selected variables as inputs and outputs cannot take any negative values or undergo a procedure that will allow them to be included in the analysis with non-negative values.

Assumption 3 (Convexity constraint) *The weights*$\lambda_{i}$*sum up to* 1, *i.e.,* $\overset{C}{\underset{i=1}{\sum }}\lambda_{i}=1$.

The convexity constraint is the main feature that distinguishes the BCC DEA from the CCR DEA and assumes that the model accounts for variable returns to scale (VRS) instead of constant returns to scale (CRS).

We suppose we have DMUs denoted by ${\text{DMS}}_{\text{i}}$ (i=1,…,C) representing the OECD member states. The DMUs use a set of N inputs $\text{x}=\left({\text{x}}_{1},\ldots ,{\text{x}}_{\text{n}}\right)\in {\text{R}}_{+}^{\text{N}}$, which proxy for the helices, to produce a single output $\text{y}\in {\text{R}}_{+}$, which measures innovativeness. Given that we aim to examine how efficient are DMUs in using the inputs to produce the output, we develop an input-oriented model with the objective function

(1) $$\text{f}\left(\text{x},\text{y}\right)=\text{min ​}{\text{θ}}_{\text{i}}$$

s. t.

(2) $$\overset{\text{C}}{\underset{\text{i}=1}{\sum }}{\text{λ}}_{\text{i}}{\text{x}}_{\text{i},\text{n}}\leq {\text{θ}}_{\text{i}}{\text{x}}_{0,\text{n}},\text{​n}=1,\ldots ,\text{N}$$

(3) $$\overset{\text{C}}{\underset{\text{i}=1}{\sum }}{\text{λ}}_{\text{i}}{\text{y}}_{\text{i}}\geq {\text{y}}_{0}$$

(4) $$\overset{\text{C}}{\underset{\text{i}=1}{\sum }}{\text{λ}}_{\text{i}}=1$$

(5) $${\text{x}}_{\text{i},\text{n}},{\text{y}}_{\text{i}}\geq 0$$

(6) $${\text{λ}}_{\text{i}}\geq 0$$

where $\;\theta_{i}$ is the efficiency score, $\lambda_{i}$ are the intensity weights for the linear combination of the sampled countries and $\;\theta_{i}=\overset{C}{\underset{i=1}{\sum }}\lambda_{i}x_{i,n}/\overset{C}{\underset{i=1}{\sum }}\lambda_{i}y_{i,m}$ denotes the efficiency score. The constraint in (4) results directly from Assumption 3, while the constraints in (5) and (6) come from Assumption 2.

The efficiency measure subject to optimisation in the objective function (1) is a kind of radial efficiency with low discriminating power and indicates that a proportional factor should reduce all inputs. In order to solve this problem, we develop a non-radial model with higher discriminatory power, which breaks down the efficiency measure into efficiency scores corresponding to the different inputs. Our non-radial input-oriented model has the objective function

(7) $$\text{f}\left(\text{x},\text{y}\right)=\text{min}\frac{1}{\text{N}}\overset{\text{N}}{\underset{\text{n}=1}{\sum }}{\text{θ}}_{\text{i},\text{n}}$$

s. t.

(8) $$\overset{\text{C}}{\underset{\text{i}=1}{\sum }}{\text{λ}}_{\text{i}}{\text{x}}_{\text{i},\text{n}}={\text{θ}}_{\text{i},\text{n}}{\text{x}}_{0,\text{n}},\text{​n}=1,\ldots ,\text{N}$$

(9) $$\overset{\text{C}}{\underset{\text{i}=1}{\sum }}{\text{λ}}_{\text{i}}{\text{y}}_{\text{i}}\geq {\text{y}}_{0}$$

(10) $$\overset{\text{C}}{\underset{\text{i}=1}{\sum }}{\text{λ}}_{\text{i}}=1$$

(11) $${\text{x}}_{\text{i},\text{n}},{\text{y}}_{\text{i}}\geq 0$$

(12) $${\text{λ}}_{\text{i}}\geq 0$$

(13) $${\text{θ}}_{\text{i},\text{n}}\leq 1$$

where the estimated value of each efficiency score ${\text{θ}}_{\text{i},\text{n}}$ points out the factor by which the corresponding input n=1,…,N has to be reduced.

At the end of this section, we follow Deng et al. (2007) and introduce the following two definitions necessary to achieve relative DEA-efficiency.

Definition 1 *If the optimal program satisfies*$f\left(x,y\right)=\min\;\theta_{i}$*, then*$\mathrm{DM}U_{i}$*is weakly DEA-efficient.*

This definition tells that the $\;\theta_{i}=1$ is the efficient score obtained from the optimisation. In other words, this means that a weakly DEA-efficient $DMU_{i}$ when $\;\theta_{i}=1$ lies on the DEA frontier. In case $\;\theta_{i}<1$, then the $\;1-\theta_{i}$ is an inefficiency margin, revealing how much the output level should be improved at the given inputs to reach efficiency.

Definition 2 *If the optimal program satisfies*Definition 1*and*Assumption 2*holds, then*$\mathrm{DM}U_{i}$*is relatively DEA-efficient.*

The importance of Definition 2 is that it gives conditions that should be satisfied in order to reach a stronger form of DEA-efficiency.

## 6 Results and discussion

In this section, we present and discuss the results of the empirical analysis. We start with the baseline TH model and then move on to its extension to a QH model.

### 6.1 Triple helix model

In the baseline DEA model, we use three input variables: the Education Index, the industrial value added and the R&D expenditure, and the total number of patent applications as an output variable. Given that there are missing values for some countries and substantial discrepancies in the results from year to year, we use the country means over the period 2006–2018 as values for the variables in the model to estimate efficiency scores for each country over the entire analysed period.

The efficiency margins from the DEA analysis of the baseline triple helix model are reported in Table 5 of Appendix 3. OECD member states have an inefficiency margin of 0.119 on average as indicated by the radial model, which points out that countries could achieve the same level of innovativeness if they decrease the inputs by a proportional factor of 11.9 per cent and make a better allocation. Greece, Japan, Luxembourg, Mexico, New Zealand, South Korea, Turkey, and the United States are on the efficiency frontier, implying that they do not have any room to reach the same level of innovativeness by decreasing the inputs. On the other hand, the largest inefficiency margin has been calculated for Norway (25.1 per cent), the Czech Republic (23.7 per cent), and Ireland (22.7 per cent).

Nonetheless, these estimates have the shortcoming that they assume a proportional factor by which all inputs can be reduced, which is not highly likely in practice, so we run a non-radial model to decompose the inefficiency margin by different scores for each input. Our estimates from the non-radial model show that the average total inefficiency margin for all OECD countries is 0.253, which is substantially higher than the calculated score from the radian model, with all countries recording a non-increasing efficiency. All countries have worsened their efficiency, except for five lying on the efficiency frontier out of eight in the radian model. They are Greece, Luxembourg, Mexico, New Zealand, and Turkey. The country with the largest total inefficiency score is Finland (41.8 per cent), followed by Switzerland (41.0 per cent), and Germany (40.8 per cent). Regarding the factors of change for the inputs, the results reveal that R&D expenditure has the largest room for decrease with a mean score of 0.464, which means that countries could decrease this input by 46.4 per cent on average and still keep the same level of innovations. Individual value added and the Education Index have mean inefficiency scores of 0.238 and 0.057, respectively. Amongst respective countries, Sweden has the largest inefficiency score for R&D expenditure (75.5 per cent), the Czech Republic for industrial value added (54.4 per cent), and Poland for the Education Index (19.6 per cent).

### 6.2 Quintuple helix model

In the recent decade, the evolution of the triple helix innovation model has seen extensions to a quadruple and a quintuple model to provide a more overarching set-up for modelling innovation (see Galvao et al., 2019). The quadruple helix model adds civil society as the fourth component in that context. The quintuple model further captures the environment as an important input in modelling innovation (Carayannis et al., 2012). In this subsection, we make a step forward to incorporating civil society and the environment as additional inputs, so we develop a QNH model in which the Civil Society Participation index is used as an input variable for society and the level of CO_2_ emissions per capita as an input variable for the environment.

Estimating the radial quintuple helix model shows an average inefficiency score of 0.058, which halves the mean inefficiency from the TH model, indicating that the OECD member states have a better allocation of five inputs to yield the given level of innovations. Noticeably, the number of countries lying on the efficiency frontier has increased to ten, with Portugal and Slovakia joining the DEA-efficient countries from the TH model, while Ireland and Denmark are very close to the frontier with an inefficiency score of 0.001 and 0.007, respectively. Lower inefficiency scores compared to the radial TH model have been found for all countries, with Germany recording the highest inefficiency of 19.7 per cent.

In the same way as with the TH model, the estimates from the non-radial QNH point out a higher inefficiency with a score of 0.218 on average. All countries have likewise worsened their inefficiency in this set-up, with seven DEA-efficient countries out of ten in the radian model. The factors of change for the three inputs included in the TH model are similar and commensurably lower given the mean total inefficiency score. For instance, a mean inefficiency score of 0.399 is estimated for the R&D expenditure, 0.145 for the industrial value added and 0.027 for the Education Index. Furthermore, the estimated scores for the added inputs show that civil society participation could be reduced by 8.6 per cent to achieve the same innovation level. In contrast, the CO_2_ emissions per capita could be reduced by 43.1 per cent, which indicates that OECD countries have room to increase environmental protection by reducing emissions and retaining the level of innovativeness. Of the individual countries, the largest inefficiency score for CO_2_ emissions per capita of 0.987 is estimated for the United States, implying that the country is in a huge excess of this input while supporting innovations.

## 7 Concluding remarks

The advent of knowledge-based economies with a particular focus on innovation has recently garnered attention in analysing sustainable economic growth and development. In that context, the TH model has become a widely studied conceptual framework to research innovation. Nonetheless, only a few studies empirically test and verify the importance of innovation actors in the system. In order to fill this gap in the economic and business literature, we acknowledge the usefulness and the relatively simple set-up of the DEA as an input-output framework for measuring efficiency and employ this methodological and empirical framework to examine how well countries utilise their resources to boost innovation.

The findings from our analysis show that the OECD countries generally have opportunities to reduce their inputs to keep the same level of innovativeness. In the radian models, we find that inputs could be reduced by a proportional factor of 11.9 per cent on average. In comparison, the factor of change in the QNT model is estimated to be 5.8 per cent on average, which indicates that countries have a better allocation of inputs in a model with more helices. From the estimates of the non-radian models, we conclude that there is increased inefficiency when different efficiency scores are estimated for the inputs. Of the individual inputs, we estimate the largest factor of change of 43.1 per cent on average for the CO_2_ emissions, implying that OECD member states could retain the level of the same innovation with strengthened environmental protection. We also find substantial mean factors of change for the industrial value added and the R&D expenditure, whereas those for the Education Index and the Civil Society Participation are significantly lower.

However, our approach to linking the TH model and the DEA framework to produce a sensible efficiency analysis is subject to some limitations that could be addressed in future research. Firstly, the limited availability of data makes it difficult to opt-in for a wide range of variables, increasing the comprehensiveness of the model. Secondly, the TH model and its extensions are complex frameworks for linking the composite helices with innovations. It is often hard to select input variables that are the best proxies for the helices and their interactions. In addition, the inclusion of the environment as the fifth helix is not generally accepted, and different authors propose different areas which need to be integrated into the model. Thirdly, the assumption that all helices are equally powerful in innovation is contestable given their state and interaction.

The possible areas of related future research would be intensely dependent on the capability to solve these limitations and run an analysis that will give more convincing results. To that point, a more dynamic analysis with an estimation of annual efficiency scores and the application of a DEA window analysis and the development of non-radial models in which the input variables will have different weights can potentially find their place in future research.

## Declarations

### Author contribution statement

Filip Fidanoski; Kiril Simeonovski; Tamara Kaftandzieva; Marina Ranga; Leo-Paul Dana; Milivoje Davidovic; Magdalena Ziolo; Bruno S. Sergi: Conceived and designed the experiments; Performed the experiments; Analyzed and interpreted the data; Contributed reagents, materials, analysis tools or data; Wrote the paper.

### Funding statement

This work was supported by the project of the National Science Center Poland (NCN) OPUS16 2018/31/B/HS4/00570.

### Data availability statement

Data will be made available on request.

### Declaration of interest's statement

The authors declare no conflict of interest.

### Additional information

The views and results elaborated in this paper are those of the authors and do not necessarily represent the official policy or position of the Joint Research Centre, European Commission, or any other organisations where the authors are working.

## Figures and Tables

**Table 2:** List of sampled countries.

Country	Country code	Geographic region	Year of accession
Canada	CAN	North America	1961
United States	USA	North America	1961
United Kingdom	GBR	Europe	1961
Denmark	DNK	Europe	1961
Iceland	ISL	Europe	1961
Norway	NOR	Europe	1961
Turkey	TUR	Europe	1961
Spain	ESP	Europe	1961
Portugal	PRT	Europe	1961
France	FRA	Europe	1961
Ireland	IRL	Europe	1961
Belgium	BEL	Europe	1961
Germany	DEU	Europe	1961
Greece	GRC	Europe	1961
Sweden	SWE	Europe	1961
Switzerland	CHE	Europe	1961
Austria	AUT	Europe	1961
Netherlands	NLD	Europe	1961
Luxembourg	LUX	Europe	1961
Italy	ITA	Europe	1962
Japan	JPN	Asia	1964
Finland	FIN	Europe	1969
Australia	AUS	Oceania	1971
New Zealand	NZL	Oceania	1973
Mexico	MEX	North America	1994
Czech Republic	CZE	Europe	1995
Hungary	HUN	Europe	1996
Poland	POL	Europe	1996
South Korea	KOR	Asia	1996
Slovakia	SVK	Europe	2000

Source: OECD.

**Table 3:** Summary statistics for the variables used in the DEA analysis over time.

Year	Measure	Variable					
		EDI	IVA	RDE	CSP	CO2	PAT
2006	Mean	1.7	26.2	80.9	74.0	11.4	63.6
	Min	0.4	13.6	54.8	59.6	3.8	0.0
	Max	3.5	39.7	91.1	99.8	26.4	343.1
	St. Dev.	0.9	5.6	8.4	22.9	5.2	88.8
2007	Mean	1.7	25.9	81.3	74.2	11.3	62.2
	Min	0.4	14.9	55.7	60.8	4.1	2.7
	Max	3.3	37.2	91.5	99.6	25.1	354.3
	St. Dev.	0.9	5.4	8.2	23.0	5.2	89.1
2008	Mean	1.9	25.7	82.0	73.9	11.0	58.9
	Min	0.4	13.4	56.4	62.4	3.9	3.4
	Max	3.5	40.3	91.9	99.3	24.7	347.8
	St. Dev.	0.9	5.8	8.2	23.0	5.0	85.2
2009	Mean	1.9	24.1	82.5	73.9	10.3	55.1
	Min	0.5	11.5	58.4	62.1	4.0	3.8
	Max	3.7	34.3	92.3	99.5	22.9	331.6
	St. Dev.	0.9	5.4	7.9	23.1	4.9	79.1
2010	Mean	1.9	24.2	83.1	74.5	10.6	54.4
	Min	0.5	11.4	62.3	56.1	3.9	4.6
	Max	3.7	34.8	92.5	99.6	23.7	343.3
	St. Dev.	0.9	5.6	7.5	23.2	5.0	80.7
2011	Mean	1.9	24.4	83.7	74.3	10.2	54.3
	Min	0.5	11.2	63.8	54.0	4.1	4.8
	Max	3.6	37.0	93.0	99.6	22.9	358.3
	St. Dev.	0.9	6.0	7.2	23.4	4.9	82.3
2012	Mean	2.0	24.1	84.1	74.4	10.0	56.3
	Min	0.4	10.7	65.0	53.0	4.2	3.8
	Max	3.9	36.8	93.3	99.7	21.9	376.3
	St. Dev.	1.0	6.0	7.0	23.5	4.9	86.0
2013	Mean	1.9	23.8	85.0	72.1	9.9	56.8
	Min	0.4	10.9	64.8	46.6	4.2	3.9
	Max	4.0	35.6	93.1	99.8	20.3	405.7
	St. Dev.	0.9	5.7	6.9	23.6	4.8	89.5
2014	Mean	2.0	23.8	85.6	72.1	9.6	57.2
	Min	0.4	10.5	65.5	44.5	4.0	4.3
	Max	4.1	34.2	93.6	99.7	19.3	414.4
	St. Dev.	0.9	5.9	6.8	23.7	4.7	90.9
2015	Mean	2.0	24.1	85.8	71.8	9.6	56.5
	Min	0.4	11.0	66.6	44.7	4.1	0.0
	Max	4.0	38.2	94.0	95.0	19.1	418.9
	St. Dev.	0.9	6.3	6.6	23.2	4.6	90.6
2016	Mean	1.9	23.7	86.1	71.1	9.6	57.0
	Min	0.4	11.7	67.8	33.6	4.1	3.1
	Max	4.0	37.0	94.6	94.8	19.6	407.7
	St. Dev.	0.9	6.0	6.6	23.7	4.6	88.9
2017	Mean	2.0	23.8	86.3	71.0	9.7	57.4
	Min	0.3	11.2	67.8	33.4	4.1	2.8
	Max	4.3	35.8	94.6	96.4	20.0	398.7
	St. Dev.	0.9	6.1	6.6	23.5	4.5	88.0
2018	Mean	2.0	24.3	86.3	70.1	9.6	55.4
	Min	0.3	11.8	68.4	30.7	3.9	2.2
	Max	4.5	36.8	94.6	94.9	20.0	406.9
	St. Dev.	1.0	6.3	6.6	23.5	4.6	88.3
All	Mean	1.9	24.5	84.1	72.9	10.2	57.3
	Min	0.3	10.5	54.8	30.7	3.8	0.0
	Max	4.5	40.3	94.6	99.8	26.4	418.9
	St. Dev.	0.9	5.8	7.4	23.0	4.8	85.5

**Table 4:** Country means for the variables used in the DEA analysis over the entire period.

Year	Variable					
	EDI	IVA	RDE	CSP	CO2	PAT
Australia	2.0	25.1	91.2	80.1	18.0	118.8
Austria	2.8	25.9	83.6	86.7	8.5	29.2
Belgium	2.2	20.4	87.9	83.8	14.5	8.1
Canada	1.8	26.9	86.2	78.3	18.7	106.9
Czech Republic	1.6	33.6	86.6	67.8	10.9	8.7
Denmark	2.9	20.8	91.1	95.0	8.3	30.5
Finland	3.2	25.6	89.5	93.3	10.1	32.0
France	2.2	18.0	79.4	81.6	6.4	25.2
Germany	2.8	26.9	93.1	93.9	11.2	77.5
Greece	0.8	15.3	80.6	74.3	8.6	5.9
Hungary	1.2	25.7	81.6	52.9	5.9	7.0
Iceland	2.3	20.8	87.9	n.a.	9.1	27.8
Ireland	1.4	29.1	88.9	77.7	8.5	12.4
Italy	1.3	21.9	78.1	79.2	7.2	13.6
Japan	3.3	28.4	81.9	68.9	11.6	271.3
Luxembourg	1.4	11.8	78.1	n.a.	21.5	37.9
Mexico	0.4	32.2	64.5	59.8	4.2	13.6
Netherlands	1.9	19.5	88.7	81.5	17.5	15.9
New Zealand	1.2	21.4	90.5	74.2	8.6	150.4
Norway	1.7	34.7	91.1	94.6	9.0	59.5
Poland	0.8	29.3	83.8	72.9	8.4	10.1
Portugal	1.3	19.7	73.0	71.8	5.9	5.8
Slovakia	0.7	30.8	80.8	67.6	7.5	4.6
South Korea	3.6	33.9	85.6	72.6	17.8	377.5
Spain	1.3	22.4	79.2	72.3	7.4	7.0
Sweden	3.3	23.1	87.9	84.3	6.0	26.6
Switzerland	3.1	25.6	87.8	97.7	5.4	25.6
Turkey	0.8	27.1	64.8	50.9	4.4	5.9
United Kingdom	1.6	18.7	89.2	80.8	8.2	36.0
United States	2.7	19.5	89.0	91.8	17.5	168.4

**Table 5:** Inefficiency margins from the DEA analysis of the triple helix model.

Model	Triple helix model				
	Radial	Non-radial			
Input	Total	Total	RDE	IVA	EDI
Country					
Australia	0.168	0.309	0.540	0.307	0.079
Austria	0.163	0.386	0.699	0.421	0.038
Belgium	0.163	0.326	0.646	0.250	0.083
Canada	0.139	0.280	0.467	0.350	0.024
Czech Republic	0.237	0.374	0.508	0.544	0.069
Denmark	0.190	0.368	0.706	0.279	0.117
Finland	0.206	0.418	0.741	0.411	0.102
France	0.070	0.247	0.496	0.245	0.000
Germany	0.217	0.408	0.699	0.404	0.121
Greece	0.000	0.000	0.000	0.000	0.000
Hungary	0.150	0.254	0.346	0.404	0.013
Iceland	0.166	0.329	0.610	0.290	0.088
Ireland	0.227	0.328	0.419	0.473	0.092
Italy	0.090	0.198	0.417	0.177	0.000
Japan	0.000	0.316	0.718	0.231	0.000
Luxembourg	0.000	0.000	0.000	0.000	0.000
Mexico	0.000	0.000	0.000	0.000	0.000
Netherlands	0.163	0.292	0.573	0.212	0.090
New Zealand	0.000	0.000	0.000	0.000	0.000
Norway	0.251	0.378	0.505	0.530	0.098
Poland	0.162	0.205	0.418	0.000	0.196
Portugal	0.020	0.094	0.282	0.000	0.000
Slovakia	0.145	0.187	0.376	0.000	0.185
South Korea	0.000	0.356	0.711	0.356	0.000
Spain	0.105	0.217	0.401	0.252	0.000
Sweden	0.181	0.393	0.755	0.339	0.083
Switzerland	0.196	0.410	0.744	0.405	0.082
Turkey	0.000	0.000	0.000	0.000	0.000
United Kingdom	0.162	0.245	0.494	0.153	0.087
United States	0.000	0.269	0.651	0.102	0.053
Average	0.119	0.253	0.464	0.238	0.057

**Table 6:** Inefficiency margins from the DEA analysis of the quintuple helix model.

Model	Quintuple helix model						
	Radial	Non-radial					
Input	Total	Total	RDE	IVA	EDI	CSP	CO2
Country							
Australia	0.124	0.363	0.540	0.307	0.079	0.073	0.816
Austria	0.100	0.250	0.651	0.000	0.000	0.185	0.416
Belgium	0.130	0.301	0.646	0.250	0.083	0.113	0.414
Canada	0.119	0.356	0.467	0.350	0.024	0.052	0.886
Czech Republic	0.121	0.270	0.556	0.082	0.067	0.002	0.646
Denmark	0.007	0.173	0.593	0.000	0.018	0.219	0.037
Finland	0.101	0.252	0.638	0.123	0.000	0.212	0.290
France	0.016	0.313	0.580	0.091	0.000	0.096	0.796
Germany	0.197	0.468	0.699	0.404	0.121	0.209	0.905
Greece	0.000	0.000	0.000	0.000	0.000	0.000	0.000
Hungary	0.000	0.000	0.000	0.000	0.000	0.000	0.000
Iceland	n.a.	n.a.	n.a.	n.a.	n.a.	n.a.	n.a.
Ireland	0.001	0.088	0.173	0.205	0.002	0.060	0.000
Italy	0.064	0.274	0.362	0.204	0.000	0.111	0.692
Japan	0.000	0.367	0.680	0.229	0.000	0.013	0.914
Luxembourg	n.a.	n.a.	n.a.	n.a.	n.a.	n.a.	n.a.
Mexico	0.000	0.000	0.000	0.000	0.000	0.000	0.000
Netherlands	0.116	0.329	0.573	0.212	0.090	0.088	0.681
New Zealand	0.000	0.000	0.000	0.000	0.000	0.000	0.000
Norway	0.082	0.235	0.545	0.152	0.097	0.275	0.106
Poland	0.150	0.239	0.000	0.438	0.034	0.000	0.723
Portugal	0.000	0.000	0.000	0.000	0.000	0.000	0.000
Slovakia	0.000	0.000	0.000	0.000	0.000	0.000	0.000
South Korea	0.000	0.393	0.688	0.349	0.000	0.033	0.897
Spain	0.081	0.261	0.373	0.271	0.000	0.001	0.663
Sweden	0.092	0.219	0.621	0.082	0.000	0.124	0.266
Switzerland	0.032	0.213	0.655	0.058	0.000	0.259	0.093
Turkey	0.000	0.000	0.000	0.000	0.000	0.000	0.000
United Kingdom	0.095	0.329	0.494	0.153	0.087	0.081	0.829
United States	0.000	0.397	0.651	0.102	0.053	0.191	0.987
Average	0.058	0.218	0.399	0.145	0.027	0.086	0.431

**Chart 1:**
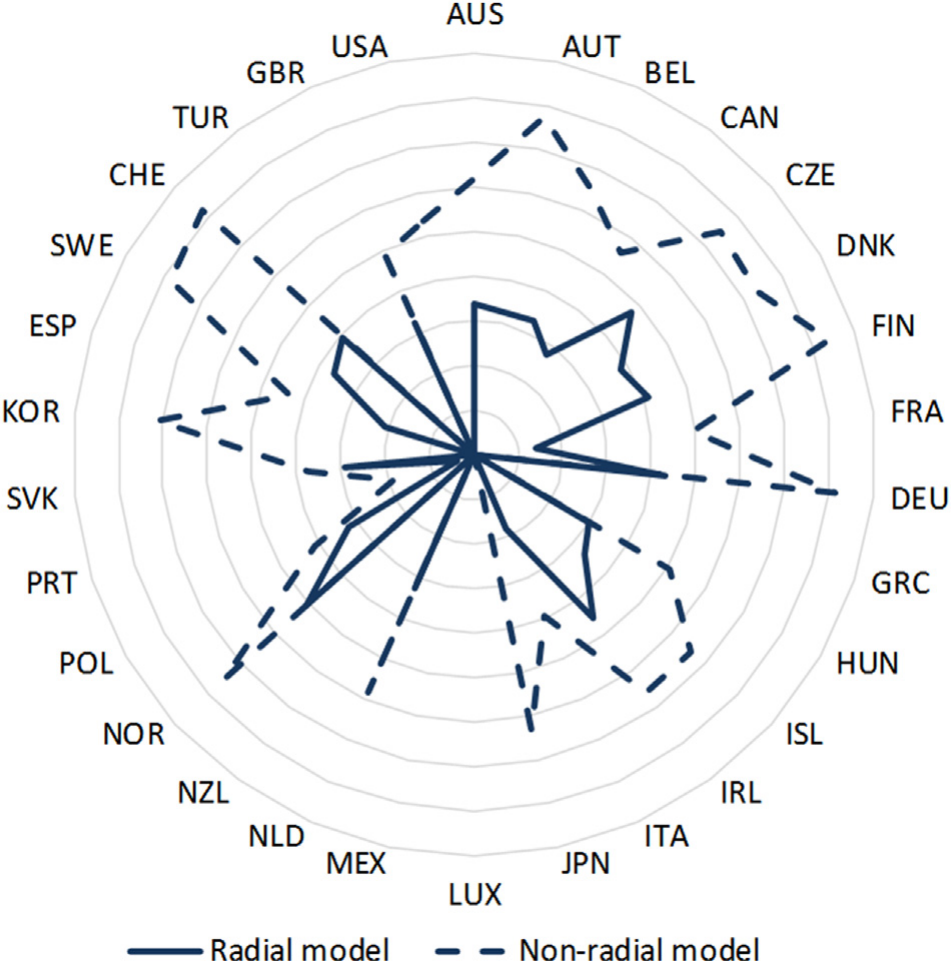
Total inefficiency margins across countries in the radial and non-radial triple helix models.

**Chart 2:**
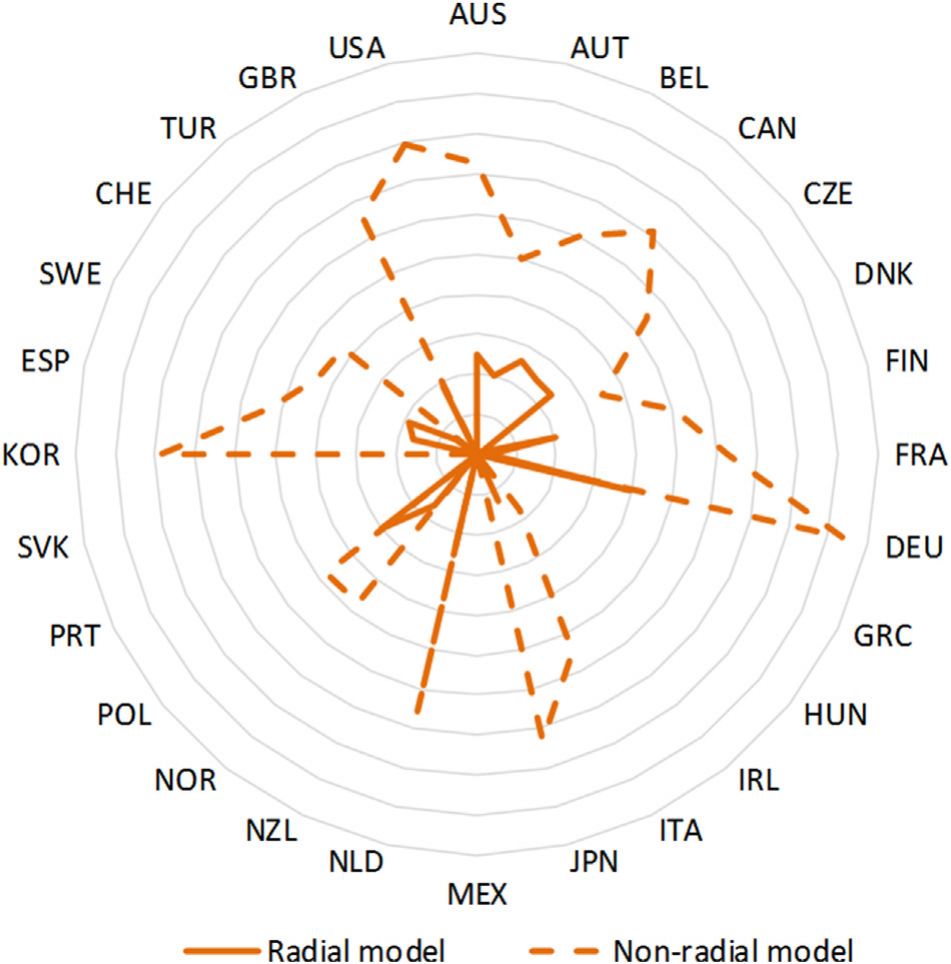
Total inefficiency margins across countries in the radial and non-radial quintuple helix models.

**Chart 3:**
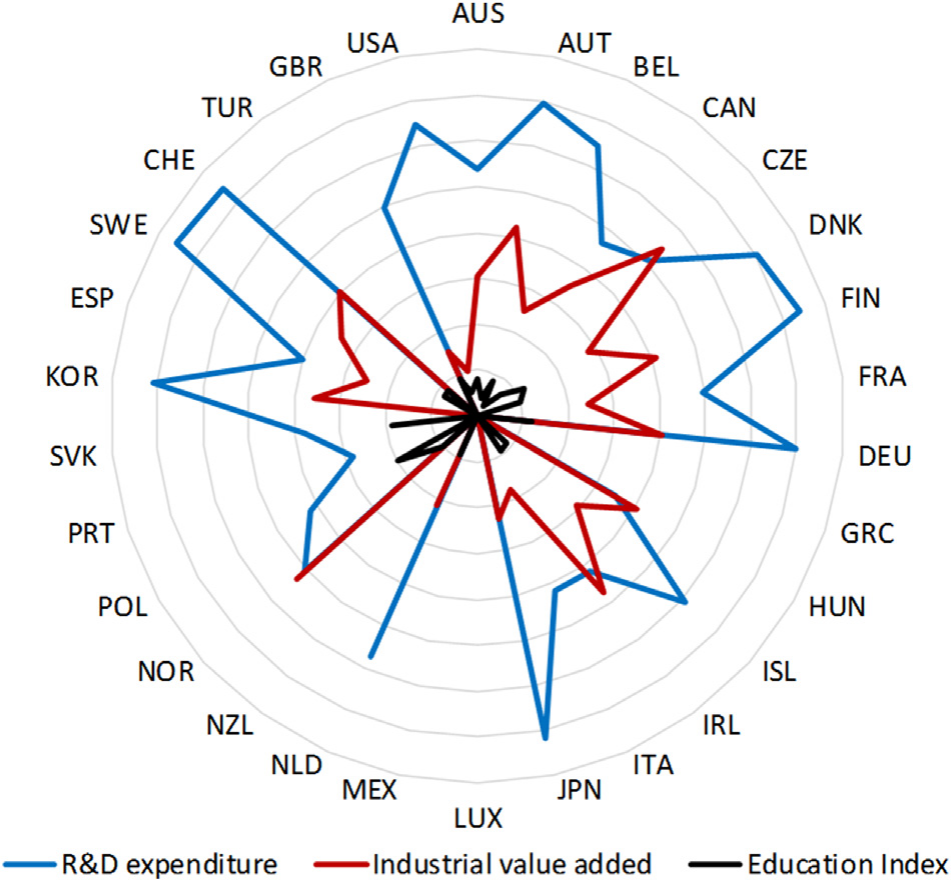
Inefficiency margins for the input variables across countries in the non-radial triple helix model.

**Chart 4:**
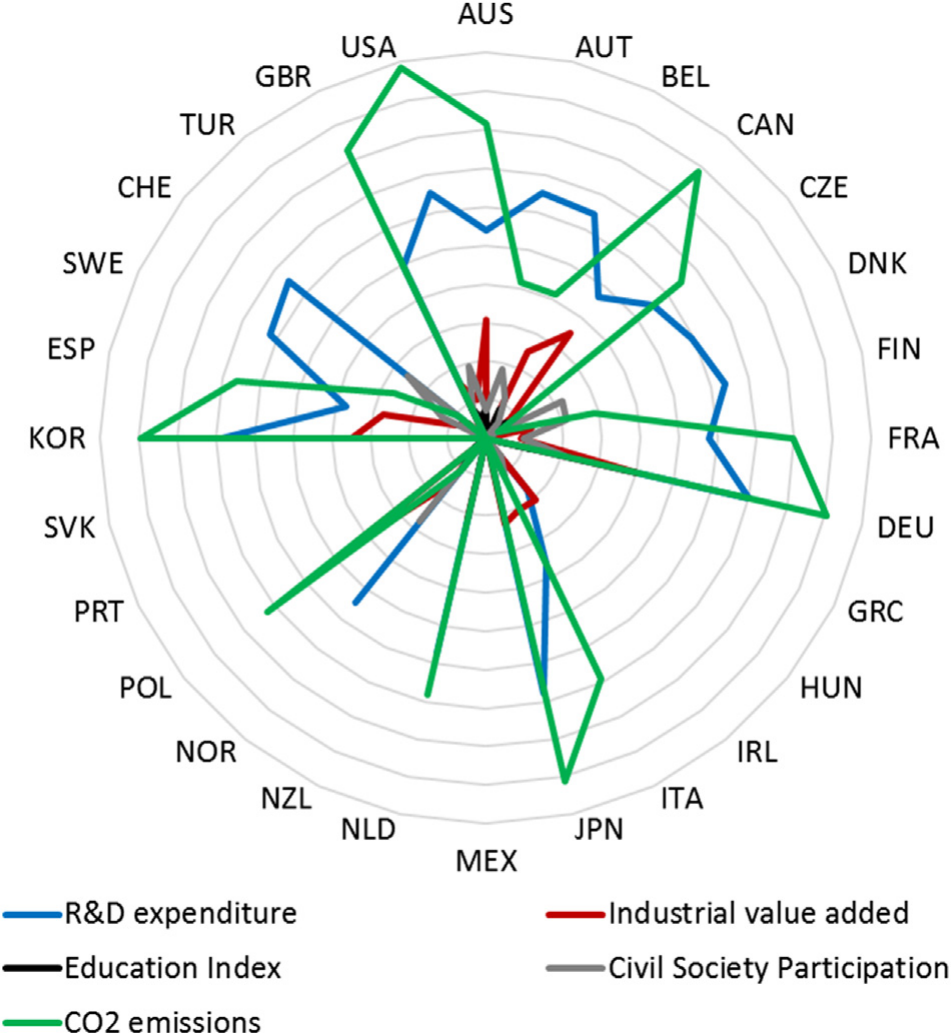
Inefficiency margins for the input variables across countries in the non-radial quintuple helix model.

## Appendix

### Appendix 1 Countries in the sample

Table 2 contains the names of all countries included in the sample with their country codes, the geographic region they belong to, and the year of accession to OECD. The year of accession was used as a principal criterion in the selection process.

### Appendix 2 Descriptive statistics

Table 3 reports summary statistics for the variables over time, while Table 4 reports the country means for the variables over the entire period.

### Appendix 3 Inefficiency margins from the DEA models

The inefficiency margins calculated as $1-{\text{θ}}_{\text{i}}$ per Definition 1 are shown in turn. Table 5 presents the margins from the DEA analysis of the triple helix model, while Table 6 reports the margins from the DEA analysis of the quintuple helix model.

### Appendix 4 Graphical representation of inefficiency scores across countries

Charts 1–4 graphically represent the inefficiency scores across countries.

## References

[bib1] Afzal M.N.I. (2014). An empirical investigation of the national innovation system (NIS) using data envelopment analysis (DEA) and the TOBIT model. Int. Rev. Appl. Econ..

[bib2] Aguilar-Barceló J.G., Higuera-Cota F. (2019).

[bib3] Anderson H.J., Stejskal J. (2019). Diffusion efficiency of innovation among EU member states: a data envelopment analysis. Economies.

[bib4] Asheim B.T., Coenen L. (2005). Knowledge bases and regional innovation systems: comparing Nordic clusters. Res. Pol..

[bib5] Audretsch D.B., Link A.N. (2012). Entrepreneurship and innovation: public policy frameworks. J. Technol. Tran..

[bib6] Audretsch D.B., Hülsbeck M., Lehmann E.E. (2011). Regional competitiveness, university spillovers and entrepreneurial activity. Small Bus. Econ..

[bib7] Balzat M., Hanusch H. (2004). Recent trends in research on national innovation systems. J. Evol. Econ..

[bib8] Brännback M., Krueger N., Carsrud A., Elfving J. (2008).

[bib9] Brem A., Radziwon A. (2017). Efficient Triple Helix collaboration fostering local niche innovation projects – a case from Denmark. Technol. Forecast. Soc. Change.

[bib10] Broekel T., Rogge N., Brenner T. (2018). The innovation efficiency of German regions–a shared-input DEA approach. Rev. Reg. Res..

[bib11] Cáceres-Carrasco F., Guzmán-Cuevas J. (2010). Functional and productive dependence: new characteristics for the analysis of enterprises from a macroeconomic view. Int. Enterpren. Manag. J..

[bib12] Cai Y. (2011).

[bib13] Cai Y., Etzkowitz H. (2020). Theorizing the triple helix model: past, present and future. Triple Helix.

[bib14] Cai Y., Liu C., Preto M.T., Daniel A., Teixeira A. (2020). Examining the Role of Entrepreneurial Universities in Regional Development.

[bib15] Cai Y., Ferrer B.R., Lastra J.L.M. (2019). Building university–industry Co-innovation networks in transnational innovation ecosystems: towards a transdisciplinary approach of integrating social sciences and artificial intelligence. Sustainability.

[bib16] Carayannis, Elias G., Barth Thorsten D., Campbell David F.J. (2012). The Quintuple Helix innovation model: global warming as a challenge and driver for innovation. J. Innovat. Entrepren..

[bib17] Carayannis E.G., Grigoroudis E., Goletsis Y. (2016). A multilevel and multistage efficiency evaluation of innovation systems: a multiobjective DEA approach. Expert Syst. Appl..

[bib18] Chen K., Guan J. (2012). Measuring the efficiency of China's regional innovation systems: application of network data envelopment analysis (DEA). Reg. Stud..

[bib19] Cooke P. (2005). Regional asymmetric knowledge capabilities and open innovation: exploring Globalization 2’A new model of industry organization. Res. Pol..

[bib20] Cooper W.W., Seiford L.M., Zhu J. (2011).

[bib21] Cullmann A., Schmidt-Ehmcke J., Zloczysti P. (2011). R&D efficiency and barriers to entry: a two-stage semi-parametric DEA approach. Oxf. Econ. Pap..

[bib22] De la Vega I., Puente J.M., Sanchez R.M. (2019). The collapse of Venezuela vs. the sustainable development of selected South American Countries. Sustainability.

[bib23] Deng C.G., Liu T., Wu J. (2007). Efficiency analysis of China’s commercial banks based on DEA: negative output investigation. China-USA Bus. Rev..

[bib24] Dosi G., Llerena P., Labini M.S. (2006). The relationships between science, technologies and their industrial exploitation: an illustration through the myths and realities of the so-called European Paradox. Res. Pol..

[bib25] Etzkowitz H. (2002).

[bib26] Etzkowitz H. (2003). Innovation in innovation: the Triple Helix of university- industry-government relations. Soc. Sci. Inf..

[bib27] Etzkowitz H. (2003). Research groups as quasi-firms: the invention of the entrepreneurial university. Res. Pol..

[bib28] Etzkowitz H. (2008).

[bib29] Etzkowitz H., Dzisah J. (2008). Triple Helix circulation: the heart of innovation and development. EASST Rev..

[bib30] Etzkowitz H., Klofsten M. (2005). The innovating region: toward a theory of knowledgebased regional development. R&D Manag..

[bib31] Etzkowitz H., Leydesdorff L. (2000). The dynamics of innovation: from national systems and Mode 2 to a Triple Helix of university-industry-governmental relations. Res. Pol..

[bib32] Etzkowitz H., Zhou C. (2017).

[bib33] Etzkowitz H., Dzisah J., Ranga M., Zhou C. (2007). The Triple Helix model of innovation. university – industry – government interaction. Asia Pac. Tech Monitor.

[bib34] Fadeyi O., Maresova P., Stemberkova R., Afolayan M., Adeoye F. (2019). Perspectives of University-Industry technology transfer in African emerging economies: evaluating the Nigerian scenario via a data envelopment approach. Soc. Sci..

[bib35] Feldman M.P., Kelley M.R. (2006). The ex-ante assessment of knowledge spillovers: government R&D policy, economic incentives and private firm behavior. Res. Pol..

[bib36] Galindo-Martin M.-A., Méndez-Picazo M., Alfaro-Navarro J. (2010). Entrepreneurship, income distribution and economic growth. Int. Enterpren. Manag. J..

[bib37] Galvao A., Mascarenhas C., Marques C., Ferreira J., Ratten V. (2019). Triple helix and its evolution: a systematic literature review. J. Sci. Technol. Pol. Manag..

[bib38] Geoghegan W., O'Kane C., Fitzgerald C. (2015). Technology transfer offices as a nexus within the triple helix: the progression of the university's role. Int. J. Technol. Manag..

[bib39] Gibbons M., Johnston R. (1974). The role of science in technological innovation. Res. Pol..

[bib40] Lafuente E., Szerb L., Acs Z.J. (2016). Country level efficiency and national systems of entrepreneurship: a data envelopment analysis approach. J. Technol. Tran..

[bib41] Lam A. (2008).

[bib42] Leydesdorff L., Meyer M. (2003). The Triple Helix of university-industry- government relations. Scientometrics.

[bib43] Leydesdorff L., Zawdie G. (2010). The triple helix perspective of innovation systems. Technol. Anal. Strat. Manag..

[bib44] Lundberg H. (2013). Triple Helix in practice: the key role of boundary spanners. Eur. J. Innovat. Manag..

[bib45] Martinelli A., Meyer M., von Tunzelmann N. (2008). Becoming an entrepreneurial university? A case study of knowledge exchange relationships and faculty attitudes in a medium sized, research-oriented university. J. Technol. Tran..

[bib46] Martynovich M. (2011).

[bib47] McAdam R., Miller K., McAdam M., Teague S. (2012). The development of university technology transfer stakeholder relationships at a regional level: lessons for the future. Technovation.

[bib48] Molas-Gallart J., Castro-Martínez E. (2007). Ambiguity and conflict in the development of ‘third mission’ indicators. Res. Eval..

[bib50] Onida F., Malerba F. (1989). R&D cooperation between industry, universities and research organizations in Europe. Technovation.

[bib51] O’Shea R.P., Allen T.J., Morse K.P., O’Gorman C., Roche F. (2007). Delineating the anatomy of an entrepreneurial university: the Massachusetts Institute of Technology experience. R&D Management.

[bib52] Pan T.W., Hung S.W., Lu W.M. (2010). DEA performance measurement of the national innovation system in Asia and Europe. Asia Pac. J. Oper. Res..

[bib53] Prokop V., Stejskal J. (2017). European Conference on Knowledge Management.

[bib54] Ranga M., Etzkowitz H. (2013). Triple helix systems: an analytical framework for innovation policy and practice in the knowledge society. Ind. High. Educ..

[bib55] Saad M., Zawdie G., Malairaj C. (2008). The Triple Helix strategy for universities in developing countries: the experiences in Malaysia and Algeria. Sci. Publ. Pol..

[bib56] Sarpong D., AbdRazak A., Alexander E., Meissner D. (2017). Organizing practices of university, industry and government that facilitate (or impede) the transition to a hybrid triple helix model of innovation. Technol. Forecast. Soc. Change.

[bib58] Smith H.L., Bagchi-Sen S. (2010). Triple Helix and regional development: a perspective from Oxfordshire in the UK. Technol. Anal. Strat. Manag..

[bib59] Strand Ø., Ivanova I., Leydesdorff L. (2017). Decomposing the triple-helix synergy into the regional innovation systems of Norway: firm data and patent networks. Qual. Quantity.

[bib60] Švarc J. (2014). A Triple Helix systems approach to strengthening the innovation potential of the Western Balkan countries. Int. J. Transit. Innovat. Syst..

[bib61] Tarnawska K., Mavroeidis V. (2015). Efficiency of the knowledge triangle policy in the EU member states: DEA approach. Triple Helix.

[bib62] Tuunainen J. (2005). Contesting a hybrid firm at a traditional university. Soc. Stud. Sci..

[bib63] Viale R., Etzkowitz H. (2005). Fifth Triple Helix Conference.

[bib64] Zabala-Iturriagagoitia J.M., Voigt P., Gutiérrez-Gracia A., Jiménez-Sáez F. (2007). Regional innovation systems: how to assess performance. Reg. Stud..

[bib65] Zemtsov S., Kotsemir M. (2019). An assessment of regional innovation system efficiency in Russia: the application of the DEA approach. Scientometrics.

[bib66] Zhou C., Etzkowitz H. (2021). Triple helix twins: a framework for achieving innovation and UN sustainable development goals. Sustainability.

[bib67] Zhuang T., Zhou Z., Li Q. (2021). University-industry-government triple helix relationship and regional innovation efficiency in China. Growth Change.

